# Platelet aggregation but not activation and degranulation during the acute post-ischemic reperfusion phase in livers with no underlying disease

**DOI:** 10.18053/jctres.201502.001

**Published:** 2015-09-13

**Authors:** Rowan F. van Golen, Katarzyna M. Stevens, Pina Colarusso, Hartmut Jaeschke, Michal Heger

**Affiliations:** 1 Department of Experimental Surgery, Academic Medical Center, University of Amsterdam, Amsterdam, The Netherlands; 2 Live Cell Imaging Facility, Snyder Institute for Chronic Diseases, University of Calgary, Calgary, Alberta, Canada; 3 Department of Pharmacology, Toxicology and Therapeutics, University of Kansas Medical Center, Kansas City, USA

**Keywords:** hepatic ischemia/reperfusion injury, sterile inflammation, platelet-neutrophil interactions, CD62P or P-selectin

## Abstract

**Background:**

Platelets and P-selectin (CD62P) play an unequivocal role in the pathology of hepatic ischemia/reperfusion (I/R) injury. Inhibition or knock-out of P-selectin or immunodepletion of platelets results in amelioration of post-ischemic inflammation, reduced hepatocellular damage, and improved survival. However, P-selectin expression on platelets and endothelial cells, which concurs with platelet activation, has never been clearly demonstrated in I/R-subjected livers.

**Aims:**

To determine whether platelets become activated and degranulate in the acute phase of liver I/R and whether the platelets interact with neutrophils.

**Methods:**

Hepatic I/R was induced in male C57BL/6J mice (N = 12) using 37.5-min ischemia time. Platelets, endothelial cells, and neutrophils were fluorescently labeled by systemic administration of non-blocking antibodies. Cell kinetics were monitored by intravital spinning disk confocal microscopy during 90 min of reperfusion. Image analysis and quantification was performed with dedicated software.

**Results:**

Platelets adhered to sinusoids more extensively in post-ischemic livers compared to livers not subjected to I/R and formed aggregates, which occurred directly after ischemia. Platelets and endothelial cells did not express P-selectin in post-ischemic livers. There was no interaction between platelets and neutrophils.

**Conclusions:**

Platelets aggregate but do not become activated and do not degranulate in post-ischemic livers. There is no platelet-neutrophil interplay during the early reperfusion phase in a moderate model of hepatic I/R injury. The mechanisms underlying the biological effects of platelets and P-selectin in this setting warrant further investigation.

**Relevance for patients:**

I/R in surgical liver patients may compromise outcome due to post-ischemic oxidative stress and sterile inflammation. Both processes are mediated in part by platelets. Understanding platelet function during I/R is key to developing effective interventions for I/R injury and improving clinical outcomes.

## 1. Introduction

In the last decade the paradigm of platelet function has expanded from primary hemostasis to also include intravascular redox signaling and sterile inflammation. Inasmuch as both oxidative stress and a sterile immune response are prominent hallmarks of hepatic ischemia/reperfusion (I/R) injury [1,2], the role of platelets has been studied in the context of I/R damage and post-ischemic liver repair. The main findings of these studies are summarized in Table 1.

**Table 1.** Summary of in vivo studies on the role of P-selectin (CD62P), platelets, and leukocytes in warm hepatic ischemia/reperfusion injury. Data reported versus controls.

Ref. Species	Major findings
[3] RAT	Animal model: 90 min of total hepatic ischemia, 4 h-7 d of reperfusion Administration of anti-CD62P antibodies improved survival and reduced post-ischemic liver damage Anti-CD62P antibodies reduced post-ischemic neutrophil adhesion and migration
[4] RAT	Animal model: 45 min of total hepatic ischemia, 6 h of reperfusion Administration of PSGL-1 reduced post-ischemic liver damage (AST, histology) and inflammation (MPO)
[5] MOUSE	Animal model: 20 min left lateral lobe ischemia, 2-24 h of reperfusion Anti-CD62P antibodies and CD62P knock-out (CD62P^-/-^) reduced the extent of post-ischemic leukocyte recruitment and rolling in sinusoidal venules (IFM)
[6] MOUSE	Animal model: 30 min left lateral lobe ischemia, 20 min – 24 h of reperfusion CD62P expression increased after ischemia (measured by amount of intrahepatic radiolabeled anti-CD62P antibody accumulation) Extent of post-ischemic liver damage (AST, ALT, LDH, histology) was reduced in CD62P^-/-^animals
[7] MOUSE	Animal model: 30-120 min of partial (40%) liver ischemia, 1-24 h of reperfusion Post-ischemic liver damage (AST, ALT, histology) and adhesion of PMN leukocytes was reduced in CD62P^-/-^animals and survival was improved (histology) Intrahepatic platelet aggregates in CD62P^-/-^animals were reduced after 90 min ischemia but increased in CD62P^-/-^animals after 120 min ischemia (CD9 immunostaining of cryosections) ---------------------------------------------------------------------------------- ---------------------------------------- ---------------------------------------- *NOTE: platelet activation status was not assessed*
[8] MOUSE	Animal model: 30 min left lateral lobe ischemia, 30-120 min of reperfusion Extent of post-ischemic leukocyte rolling, saltation, and adhesion in sinusoidal venules was reduced following administration of anti-CD62P antibodies and in CD62P^-/-^animals (IFM)
[9] MOUSE	Animal model: 90 min of partial (70-80%) liver ischemia, 3 h of reperfusion Post-ischemic liver damage (ALT) and inflammation (MPO) were reduced in CD62P^-/-^ animals Post-ischemic MIP-1 and MIP-2 levels were lower in CD62P^-/-^ animals
[10] MOUSE	Animal model: 90 min of partial liver ischemia, 1.5-6 h of reperfusion No differences in post-ischemic liver damage (ALT) between WT and CD62P^-/-^/ICAM-1^-/-^ mice CD62P^-/-^/ICAM-1^-/-^ animals exhibited more extensive hepatic neutrophil influx following I/R (histology)
[11] MOUSE	Animal model: 90 min left lateral lobe ischemia, 60 min of reperfusion I/R induced platelet adhesion in peri-sinusoidal arterioles, sinusoids, and post-sinusoidal venules (IFM) Post-ischemic platelet adhesion was reduced in ICAM-1^-/-^ and anti-fibrinogen antibodies-treated mice (IFM) ICAM-1^-/-^but not anti-fibrinogen antibodies-treated mice exhibited reduced leukocyte adhesion (IFM) Post-ischemic fibrinogen deposition in in peri-sinusoidal arterioles, sinusoids, and post-sinusoidal venules was abrogated in ICAM-1^-/-^ mice (IFM) Anti-fibrinogen antibodies improved sinusoidal perfusion and reduced post-ischemic liver damage (AST, ALT), apoptosis (TUNEL, caspase-3), and lipid peroxidation (TBARS) ---------------------------------------------------------------------------------- ---------------------------------------- ---------------------------------------- *NOTE: platelet activation status was not assessed* *NOTE: platelets were fluorescently labeled ex vivo (rhodamine 6G) and reinfused*
[12] MOUSE	Animal model: 90 min left lateral lobe ischemia, 60 min of reperfusion Post-ischemic platelet adhesion in peri-sinusoidal arterioles, sinusoids, and post-sinusoidal venules was reduced in CD62P^-/-^animals (IFM) CD62P-deficient platelets rolled on and adhered to post-ischemic hepatic microcirculation in a similar manner as platelets in WT animals (IFM) Platelet rolling and adhesion was abrogated in post-ischemic CD62P^-/-^ livers (IFM) Leukocyte rolling and adhesion was abrogated in post-ischemic CD62P^-/-^ livers (IFM) Sinusoidal perfusion was improved in CD62P^-/-^ livers (IFM) CD62P-deficiency was associated with reduced I/R damage (AST, ALT) and cell death (TUNEL, caspase-3, DNA fragmentation/nuclear condensation) (histology) ---------------------------------------------------------------------------------- ---------------------------------------- ---------------------------------------- *NOTE: platelet activation status was not assessed* *NOTE: platelets were fluorescently labeled ex vivo (rhodamine 6G) and reinfused*
[13] MOUSE	Animal model: 30-90 min left lateral lobe ischemia, 20-240 min of reperfusion I/R induced platelet rolling and adhesion in venules and arterioles and accumulation in sinusoids (FM) Platelet adhesion to post-sinusoidal venules correlated negatively with perfusion rate I/R induced thrombocytopenia I/R induced thrombin activation ------------------------------------------ *NOTE: platelet activation status was not assessed* *NOTE: platelets were fluorescently labeled ex vivo (rhodamine 6G) and reinfused*
[14] MOUSE	Animal model: 60 min of partial (70%) liver ischemia, 1 h-7 d of reperfusion Platelets did not contribute to I/R injury (CD41 antibody-mediated depletion and clopidogrel-mediated inhibition of platelet function, AST) Pre-ischemic platelet depletion reduced post-ischemic neutrophil infiltration Inhibition of platelet function (clopidogrel) has no effect on post-ischemic hepatic TNF-α, IL-6, IL-1β, MIP-2 levels (RT-PCR) Platelet depletion (CD41 antibodies) had no effect on post-ischemic hepatic TNF-α and IL-1β levels but reduced IL-6 and MIP-2 levels (RT-PCR) Platelet depletion (CD41 antibodies) reduced post-ischemic plasma levels of TNF-α and IL-6 (ELISA) Platelet depletion (CD41 antibodies) reduces the extent of post-ischemic (7 d) liver regeneration (PCNA and Ki-67, histology), which is mediated by platelet-derived serotonin (Tph1^-/-^mice) ---------------------------------------------------------------------------------- ---------------------------------------- ---------------------------------------- *NOTE: platelet activation status was not assessed*
[15] RAT	Animal model: 60 min of partial (70%) liver ischemia, 30 min - 7 d of reperfusion I/R induced endothelial CD62P expression (immunohistochemistry) and upregulation of hepatic CD62P mRNA (RT-PCR)
[16] RAT	Animal model: 60 min of partial (70%) liver ischemia, 30 min - 7 d of reperfusion I/R induced platelet adhesion in liver microcirculation Kupffer cell depletion (Cl_2_MDP) reduced I/R induced platelet adhesion in liver microcirculation (IFM) and the extent of leukocyte influx (histology) Post-ischemic sinusoidal endothelial cells and platelets associated with endothelial cells did not express CD62P* (immunohistochemistry on cryosections) ------------------------------------------------------------------------------------------------------------------------------------------------------------------ * No data were shown *NOTE: platelets were fluorescently labeled ex vivo (rhodamine 6G) and reinfused*
[17] RAT	Animal model: 20 min of complete liver ischemia, 30-90 min of reperfusion I/R induced platelet adhesion in liver microcirculation, which was reduced by the neutrophil elastase inhibitor sivelestat (IFM) ---------------------------------------------------------------------------------- ---------------------------------------- ---------------------------------------- *NOTE: platelet activation status was not assessed* *NOTE: platelets were fluorescently labeled ex vivo (rhodamine 6G) and reinfused*
[18] RAT	Animal model: 20 min of complete liver ischemia, 30-120 min of reperfusion I/R induced platelet adhesion in liver microcirculation (IFM) More than 50% of adherent platelets were associated with Kupffer cells (IFM) ------------------------------------------------------------------------------------------------------------------------------------------------------------------------ *NOTE: platelet activation status was not assessed* *NOTE: platelets were fluorescently labeled ex vivo (rhodamine 6G) and reinfused*
[19] RAT	Animal model: 20 min of complete liver ischemia, 30-120 min of reperfusion I/R induced platelet adhesion in liver microcirculation, which was reduced by the HO-1 inducer CoPP (IFM) More than 50% of adherent platelets was associated with Kupffer cells (IFM) The flow velocity of platelets in post-ischemic sinusoids was increased in CoPP-treated animals compared to untreated animals (IFM) ---------------------------------------------------------------------------------- ---------------------------------------- ---------------------------------------- *NOTE: platelet activation status was not assessed* *NOTE: platelets were fluorescently labeled ex vivo (rhodamine 6G) and reinfused*
[20] MOUSE	Animal model: 90 min left lateral lobe ischemia, 60 min of reperfusion I/R induced platelet rolling and adhesion in post-sinusoidal venules and accumulation in sinusoids Post-ischemic platelet adhesion in post-sinusoidal venules and sinusoids was reduced by the PAR-4 inhibitor TcY-NH2 (IFM) ------------------------------------------------------------------------------- ---------------------------------------- ---------------------------------------- *NOTE: platelet activation status was not assessed* *NOTE: platelets were fluorescently labeled ex vivo (rhodamine 6G) and reinfused*

Abbreviations (alphabetically): ALT, alanine aminotransferase; AST, aspartate aminotransferase; CD31, platelet endothelial cell adhesion molecule (PECAM-1); CD41, integrin alpha-IIb; CD49b, integrin, alpha 2 (alpha 2 subunit of VLA-2 receptor); CD62P, P-selectin; Cl_2_MDP, dichloromethylene diphosphonate; CoPP, cobalt protoporphyrin; FITC, fluorescein isothiocyanate; HO-1, heme oxygenase 1; ICAM-1, intercellular adhesion molecule-1 (CD54); IFM, intravital fluorescence microscopy; IL, interleukin; I/R, ischemia/reperfusion; LDH, lactate dehydrogenase; MIP, macrophage inflammatory protein; MPO, myeloperoxidase; mRNA, messenger ribonucleic acid; PAR-4, protease-activated receptor 4; PCNA, proliferating cell nuclear antigen; PE, phycoerythrin; PMN, polymorphonuclear; PSGL-1, P-selectin glycoprotein ligand-1 (CD162); RT-PCR, reverse transcription polymerase chain reaction; TBARS, thiobarbituric acid-reactive substances; TNF-α, tumor necrosis factor α; TUNEL, terminal deoxynucleotidyl transferase dUTP nick end labeling deoxynucleotidyl transferase dUTP nick end labeling; WT, wild-type.

In the early reperfusion phase, warm hepatic I/R is associated with exacerbated platelet rolling and adhesion in the hepatic microcirculation [B11, B12] and perturbed blood flow[B13]. This interaction is facilitated by intercellular adhesion molecule-1 (ICAM-1) [B11], fibrinogen [B11], and protease-activated receptor-4 (PAR-4) [B20], which is involved in the initiation of secondary hemostasis (coagulation) [B21]. Inhibition or knock-out of P-selectin (CD62P) or immunodepletion of platelets results in amelioration of post-ischemic inflammation [B14], reduced hepatocellular damage [B11,B12], and improved survival [B3], altogether attesting to a role of platelets and P-selectin in post-ischemic hepatopathology.

As such, Khandoga *et al*. [B11] postulated (or concluded) that, since “activated platelets are able to generate reactive oxygen radicals and nitric oxide and to release pro-inflammatory mediators, …activated platelets have the potential to induce I/R injury by both direct impact and aggravation of microcirculatory derangements.” Despite the apparent involvement of platelets in I/R injury, Woolbright and Jaeschke [B22] recently questioned whether the data available to date unequivocally corroborate a mediatory role of platelets in post- ischemic injury or platelet activation. On the basis of Table 1 it can be concluded that post-ischemic platelet activation and corollary degranulation (surface exposure of P-selectin), i.e., the trigger for the inflammatory processes, repair mechanisms, and the initiation of secondary hemostasis, have indeed never been closely investigated.

Accordingly, we have conducted several focused hepatic I/R experiments in mice using intravital spinning disk confocal microscopy and platelet labeling [B23] to elucidate the platelet activation status in the acute reperfusion phase (0-90 min) [B1]. The experiments yielded some unexpected and contradictory findings. Platelet aggregation occurred in the hepatic microcirculation during the acute reperfusion phase but was not associated with degranulation, which is necessary for the biological effects reported in literature (Table 1). Evidently, these findings have important implications on the regulatory role of platelets in hepatic I/R.

## 2. Materials and Methods

All supplementary material is indicated with a prefix ‘S.’

### 2.1. Animal model and surgery

The study was approved by the animal ethics committee of the University of Calgary (protocol#AC12-0162) and all animals were treated in accordance with the *Guide for the Care and Use of Laboratory Animals* (NIH publication 85-23, rev. 2011). Male C57BL/6J mice (N = 12, Charles River, Montreal, Quebec, Canada) weighing between 22-25 g were housed under standard laboratory conditions with ad libitum access to regular chow and water. The animals were acclimated for at least 2 d before entering the experiment.

Mice received analgesia by subcutaneous administration of buprenorphine (0.06 mg/kg, Temgesic, Schering-Plough, Kenilworth, NJ) following induction anesthesia with isoflurane (2.5% isoflurane in O_2_, 1 L/min, Forene, Abbott Laboratories, Queensborough, UK). Anesthesia was subsequently maintained with isoflurane (1.5% in O_2_, 0.5 L/min) during the experimental procedure. Body temperature was measured with a rectal temperature probe and was maintained at 37 °C with a heating pad (Fig. S1A, orange pad) connected to a self-regulating TR-200 homeothermic temperature controller (Fine Science Tools, Heidelberg, Germany). The unit automatically adjusted the temperature of the heating pad on the basis of the signal received from the rectal temperature probe. The animals were fixed dorsally onto the heating pad, which in turn was secured to a mobile microscope stage (Fig. S1A) placed on a Vibraplane optical table (Kinetic Systems, Boston, MA) for surgery and intravital microscopy.

Following a midline laparotomy, the left medial-, right medial-, and left lateral lobes were exteriorized, gently retracted cranially, and secured with a PBS-drenched gauze as described in [B24]. The liver hilus was mobilized and 70% ischemia was induced by clamping the portal and arterial blood supply with a 4 × 1-mm microvessel clip (MEHDORN, Aesculaep, Center Valley, PA) [B24]. Following 37.5-min ischemia, which is associated with moderate liver injury [B24], the clip was removed and a customized metal transabdominal stage (Home Depot, Calgary, Alberta, Canada) was placed over the animal’s abdomen (Fig. S1A) as described in [B25]. The transabdominal segment of the stage was convexly shaped and wrapped in gauze to ensure proper fixation of the liver lobe, elimination of breathing artifacts, and an optimal focal plane during intravital microscopy. The stage-wrapped gauze was wetted with 0.9% NaCl solution and the left lateral lobe was gently flipped onto the stage and fixed with acryl-based tissue glue (Vetbond tissue adhesive, 3M Animal Care Products, St. Paul, MN) at the distal and lateral ends of the lobe (relative to the head). Following a flush with 0.9% NaCl solution, the liver lobe was covered with saran wrap to prevent desiccation [B25]. The saran wrap was secured to the stage with a thin strip of tape (not over the liver) and the liver lobe was imaged by intravital microscopy (Fig. S1B).

### 2.2. Systemic cell labeling for intravital microscopy

Antibodies were added to sterile 0.9% NaCl solution (B. Braun Melsungen, Melsungen, Germany) to a final infusion volume of 100 μL. The used antibodies and antibody concentrations were: sinusoidal endothelial cells: rat anti-mouse CD31-PE, 10 μL of 200 μg/mL (cat.#12-0311-83, clone 390, eBioscience, San Diego, CA) or rat anti-mouse CD31-Alexa Fluor 647, 5 μL of 1000 μg/mL (cat.#16-0311-85, clone 390, eBioscience, labeled with Alexa Fluor 647 protein labeling kit, cat.#A-20173, Life Technologies, Carlsbad, CA); resting platelets: hamster anti-mouse CD49b-Alexa Fluor 647, 7 μL of 500 μg/mL (cat.#103511, clone HMα2, Biolegend, San Diego, CA); activated platelets, rat anti-mouse CD62P-FITC, 10 μL of 500 μg/mL (cat.#553744, clone RB40.34, BD Pharmingen, Franklin Lakes, NJ); neutrophils: rat anti-mouse Ly-6G (Gr-1)-FITC, 10 μL of 500 μg/mL (cat.#108406, clone R86-8C5, BioLegend). The mixture was infused into the penile vein directly before surgery using a 1 mL insulin syringe, after which the puncture wound was sealed with an electro-surgical cauterizer. Before the liver I/R experiments, in vivo thrombus staining by the CD62P-FITC antibodies was verified in a puncture-induced thrombosis model in the murine saphenous artery (N = 2, Fig. S2).

**Figure 1.**
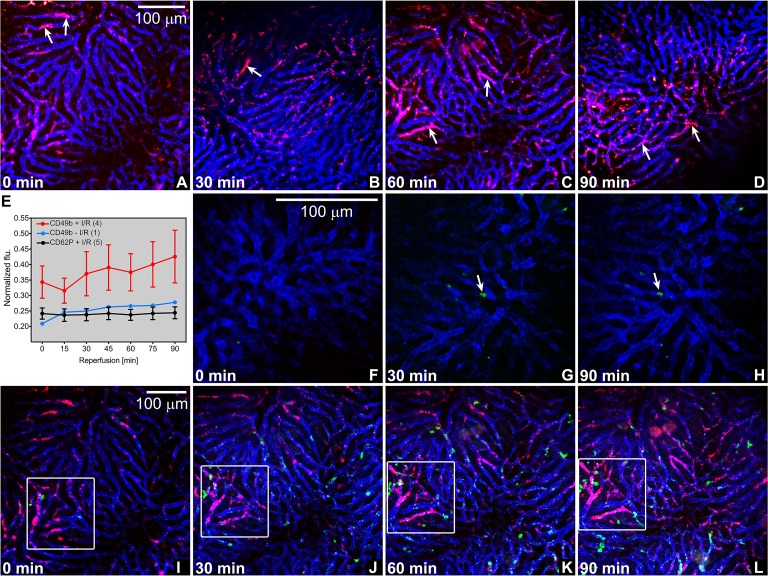
Intravital imaging of platelet aggregation and platelet activation status following hepatic I/R in mice. (A-D) Platelet aggregates (red (CD49b), arrows) in hepatic microcirculation (blue, CD31) as a function of reperfusion time (left bottom, all imaging panels). Representative panels are shown per time point, taken from the video footage of 3 animals. Scale bar applies to all panels. (E) The mean pixel intensity per fluorescence channel (*y*-axis) was quantitated for each time point (*x*-axis) for every experimental group (resting platelets following I/R (CD49b + I/R), resting platelets in sham-operated animals (CD49b – I/R), and activated platelets following I/R (CD62P + I/R)) using FiJi/ImageJ software. Platelet fluorescence (flu) was normalized to endothelial fluorescence (mean ± SEM, sample size is given in parentheses in the legend). (F-H) Absence of P-selectin staining (green, CD62P) in post-ischemic liver microcirculation (blue, CD31). Incidental P-selectin-positive foci are indicated with arrows, corresponding to the same location at different reperfusion times. (I-L) Absence of platelet (red, CD49b) and neutrophil (green, Gr1) colocalization in post-ischemic hepatic microcirculation (blue, CD31). The quadrant corresponds to the same location at different reperfusion times, whereas the time lapse series in I-L correspond to panel C. Note the gradual increase in platelet aggregation in the demarcated region in this animal.

### 2.3. Intravital microscopy

Intravital microscopy was performed with a Quorum Wave FX-X1 spinning disk confocal system that consisted of an upright Olympus IX51 microscope (Olympus Corporation, Tokyo, Japan) equipped with a Yokogawa CSU-X1 scan head (Yokogawa Electric, Tokyo, Japan), a back-thinned Hamamatsu EMCCD camera (model C9100-13, Hamamatsu Photonics, Hamamatsu City, Japan), and 491-, 561-, and 640-nm excitation lasers. The following emission filters were used for the antibody-conjugated fluorophores: 536 ± 40 nm (CD62P-FITC and Gr-1-FITC), 593 ± 40 nm (CD31-PE), and 692 ± 40 nm (CD31-Alexa Fluor 647 and CD49b-Alexa Fluor 647), respectively. The emission filters were under the control of a MAC 6000 Modular Automation Controller (Ludl Electronic Products, Hawthorne, NY). Imaging was performed with an Olympus UPlanFL-N, 10×, NA = 0.2 objective. The hardware settings were kept constant during all experiments (FITC channel: laser power 60, exposure time 80 ms, camera gain 1, camera sensitivity 209; PE channel: laser power 71, exposure time 120 ms, camera gain 1, camera sensitivity 224; Alexa Fluor 647 channel: laser power 85, exposure time 120 ms, camera gain 1, camera sensitivity 171). Image acquisition was performed under Volocity software control (Version 6.3.1, Perkin Elmer, Waltham, MA). Image acquisition was performed for 1 min at a frame rate of 11 Hz (3 fluorophores) or 13 Hz (2 fluorophores) after removal of the microvascular clip (the time interval between clip removal and image acquisition was on average 4 min) and at t = 4 + 15, 30, 45, 60, 75, and 90 min reperfusion. The abdominal cavity was hydrated via the base of the liver lobe after each image acquisition sequence with a syringe containing 0.9% NaCl solution (37 °C).

### 2.4. Image analysis

Image analysis was performed using ImageJ/FIJI (NIH, Bethesda, MD). Volocity files were imported into Fiji as colorized hyperstacks using the Bio-Formats Importer. After splitting the fluorescence channels, the total pixel intensity per frame was measured for each channel using FIJI’s automated analysis module (“analyze→measure”). The fluorescence intensity from platelets (i.e., CD62P or CD49b) was first normalized per frame to the fluorescence intensity from endothelium (i.e., CD31) to correct for loss-of-focus within the ROI during image acquisition. Subsequently, the normalized pixel intensity per frame was averaged per time point (i.e., the mean normalized pixel intensity of 11 or 13 frames was calculated for each 1-min imaging sequence). Data are presented as the mean platelet fluorescence/endothelial fluorescence ratio per time point.

## 3. Results

First, systemically labeled platelets (CD49b-Alexa Fluor 647, pan-platelet staining) adhered to sinusoids more extensively in post-ischemic livers (Fig. 1A-E) compared to livers not subjected to I/R (Fig. S3) and formed aggregates, which occurred from the very onset of reperfusion. It is therefore likely that platelet-vascular wall interactions transpired mainly during ischemia, particularly since the platelet aggregates did not notably increase during 90-min reperfusion and were not abundantly present in sham-operated animals (Fig. 1E). Second, P-selectin staining of activated platelets and endothelial cells was entirely absent in post-ischemic livers (Fig. 1F-H), indicating that neither α-granule nor Weibel-Palade body secretion from platelets and endothelial cells, respectively, occurs during ischemia and early reperfusion. Third, to investigate the interplay between platelet aggregates and neutrophils (demonstrated in [B26]), systemic staining was performed for endothelium (CD31-PE), platelets (CD49b-Alexa Fluor 647), and neutrophils (Gr-1-FITC) before the induction of I/R. Of note, platelet-neutrophil interactions are facilitated by glycoprotein Ibα (CD42b) [B26], which were not blocked by the mentioned fluorescently labeled antibodies. As shown in Fig. 1I-H, there was no notable heterotypic aggregate formation that involved platelets and neutrophils. Whereas the extent of platelet aggregate formation remained relatively stable over the reperfusion time (Fig. 1E), the influx and adhesion of neutrophils increased with reperfusion time (manuscript in preparation).

## 4. Discussion

Taken together, these data demonstrate that [a] platelets aggregate but do not become activated in post-ischemic livers and [b] there is no platelet-neutrophil interplay during the early reperfusion phase. Platelet aggregation following liver I/R in mice was visualized with spinning-disk intravital confocal microscopy using fluorescent anti-CD49b antibodies as an in vivo pan-platelet label. Although the use of anti-CD49b antibodies deters a potential interaction of platelets with certain substrates (e.g., collagen [B27]), this staining method has a high labeling efficiency, does not affect platelet phenotype [B23], and obviates the need for intricate ex vivo platelet staining procedures that may affect platelet phenotype or function [B28]. The finding that platelets adhere extensively to sinusoidal endothelium during the first 90 min of reperfusion is in line with previous intravital imaging observations in murine liver I/R models [11-13;20;29]. However, the biological significance of this phenomenon is less clear. As mentioned in the introduction, platelet aggregation has been causally linked to, e.g., microvascular perfusion defects, apoptotic cell death, vascular oxidative stress, and an inflamed endothelium [11-13;20], all alluding to a pathophysiological connection between platelets and surgery-induced hepatopathology. There are, however, important considerations that need to be kept in mind when interpreting the results from the cited studies.

The parameter that has been most extensively used to study platelet function in liver I/R is P-selectin, which is released from α-granules during platelet activation and is subsequently expressed on the platelet outer membrane leaflet to facilitate thrombosis via platelet-platelet and platelet-neutrophil interactions [B12]. Although P-selectin-deficient animals generally exhibit an attenuated liver I/R injury profile (Table 1), suggesting pathological platelet behavior, this genetic model does not differentiate between platelet and endothelial P-selectin. This distinction is crucial insofar as endothelial P-selectin enables leukocyte adhesion in postsinusoidal venules under inflammatory conditions [30-32], which could explain the hepatoprotective efficacy of a generalized P-selectin deficiency. In addition, it has been posited that anti-P-selectin therapies reduce hepatic I/R injury via protective effects on the intestinal microcirculation [B32] rather than through direct effects on the liver, which underscores that a more targeted approach is necessary to selectively explore platelet P-selectin function. In light of these considerations, it should be noted that endothelial P-selectin expression was not observed during the first 90 min of reperfusion in the current experiments (Fig. 1F-H), which might be due to the fact that leukocyte adhesion typically occurs at later stages of I/R injury (i.e., the chronic reperfusion phase [B1]) and/or the fact that sinusoidal endothelium mainly relies on ICAM-1 instead of P-selectin to immobilize chemoattracted leukocytes following hepatic I/R. The latter may also relate to the reported absence of P-selectin-containing Weibel-Palade bodies in sinusoidal endothelial cells, albeit contradictory findings on this subject have been published (discussed in [B33,B34]).

In order to properly assess platelet function under inflammatory conditions, it is imperative to also determine platelet activation status, which is a frequent omission in platelet-centered liver I/R studies ([B22], see also Table 1). Using a validated antibody-based in vivo P-selectin labeling method (Fig. S2), it was shown that liver I/R in mice triggers platelet aggregation without notable P-selectin expression (Fig. 1F-H), which deviates from the putative platelet activation paradigm. Several factors could explain this observation. First, platelets can form reversible aggregates based on integrin-fibrinogen or integrin-endothelial interactions only, which can occur independently of α-granule (i.e., P-selectin) release and does not require soluble platelet agonists such as ADP or thrombin [35-37]. Based on Fig. 1I-L, however, the reversible nature of such aggregates is entirely absent given the fact that all aggregates in the demarcated region are expanding in time over a 90-min time span. Alternatively, and more plausibly, autocrine and/or paracrine signals may terminate the platelet activation cascade before α-granule secretion takes place [B38]. Such signals can be either derived from platelets (e.g., release of tissue factor pathway inhibitor and/or protein S) or mediated by activated endothelium (e.g., through production of prostacyclin I_2_ and/or nitric oxide) [B38]. Tissue factor pathway inhibitor and protein S are localized in intracellular storage granules of platelets and endothelial cells and hence require activation-stimulated degranulation and release [39-42] to become biologically available. The lack of degranulation in aggregated platelets and endothelial cells, as evidenced by the absence of P-selectin-positive staining, therefore precludes that this mechanism was mediated by intracellular tissue factor pathway inhibitor and protein S. However, tissue factor pathway inhibitor and protein S are abundant in plasma [B43,B44] and could therefore facilitate the activation of the cascade-terminating process.

Notwithstanding the lack of P-selectin expression, Fig. 1A-D unequivocally confirm that platelets aggregate extensively during early reperfusion, leading to complete obstruction of the vascular lumen in some sinusoids. It is unclear whether this extent of platelet aggregation can occur independently of P- selectin release and corollary coagulation activation (i.e., thrombus formation). With respect to the latter, thrombosis following I/R has only been documented by one research group in rats [B29], albeit a disproportionally severe I/R injury model was employed. On the other hand, our group has shown that the coagulation cascade is activated following I/R in rats (30 min partial liver ischemia), which resulted in thrombin and fibrin formation in the acute reperfusion phase (30 min) [B45]. Thrombin and to a lesser extent fibrin [B46] induce platelet activation and aggregation, which begs the question why no P-selectin exposure was observed.

**Figure 2.**
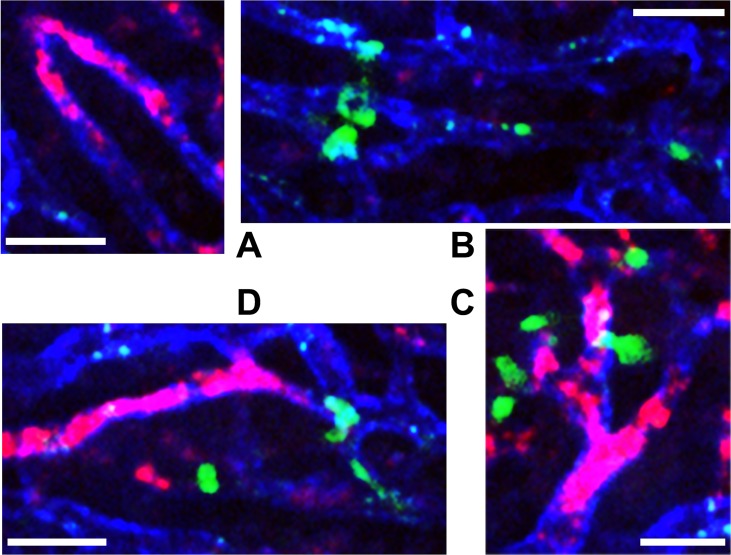
Absence of platelet-neutrophil interactions after hepatic I/R in mice. Systemic triple staining and intravital imaging of platelets (red), endothelium (blue), and neutrophils (green) was performed as described in sections 2.2 and 2.3, and [B23]. Panels A and B show close-ups of I/R-induced platelet plugs and sinusoidal neutrophil adhesion, respectively. In contrast to recent venous thrombosis literature [B26], neutrophils did not mediate the formation of platelet aggregates after I/R, as evidenced by the lack of neutrophil-platelet co-location (C, D). Scale bar = 30 μm.

Consequently, the findings in this study render the thrombosis paradigm in liver I/R injury equivocal and elusive for two reasons. First, the most important biological processes that lead to intravascular thrombosis, which include platelet aggregation, endothelial damage, and innate immune activity, are well-established liver I/R injury hallmarks. It was therefore expected that thrombosis, during which P-selectin is translocated to the outer membrane such that systemically administered antibodies can bind, would be detected. Second, local perfusion deficits have been routinely described in murine liver I/R models [B47]. However, (micro)vascular perfusion failure has been attributed to vasoconstriction, edema [B45], and consequent leukocyte plugging rather than platelet aggregation. The data presented here present an inverse scenario, in which platelet aggregates actually occlude the vascular lumen first, with an apparent minor role for neutrophils or neutrophil-platelet interactions (Fig. 2) in the acute reperfusion phase. This finding is in agreement with the paradigm that neutrophils are slow to accumulate during the first hours of reperfusion and in this stage do not (yet) contribute significantly to oxidative stress [B48]. Any effect of platelets on neutrophils, if there is one, therefore only has very limited impact on the pathophysiology of liver I/R injury.

Collectively, these findings place the biological significance of platelets in post-ischemic liver pathology in a different light. Owing to their established involvement in thrombotic and inflammatory processes, platelets are often deemed harmful by default. Recent reports, however, challenge this claim. First, it has been shown that immunodepletion of platelets using CD41 antibodies does not protect mice from hepatic I/R injury, but does delay functional liver recovery in the long run [B14]. Corroboratively, it has been postulated that platelets relay the protective signals of remote ischemic preconditioning [B49] and are essential for the liver to regenerate properly following a partial liver resection [B50,B51]. The same trend is seen in immunological platelet studies, which increasingly recognize that platelets are not merely cytokine factories, but also coordinate antimi-crobial responses in close collaboration with Kupffer cells [B52]. Insofar as an association between platelets and KCs has been repeatedly shown in murine liver I/R studies [B18,B19], these findings indicate that platelets might actually play a beneficial role in liver I/R injury.

In summary, it is concluded that platelet aggregation in post-ischemic livers does not abide by the putative thrombosis-inflammation mechanisms [B26]. How platelets aggregate without becoming activated and how they mediate the biological and immunological processes alluded to previously and in Table 1 without degranulating should be subjected to experimental scrutiny.
